# Supplementary material for: Canonical and noncanonical TGF-β signaling regulate fibrous tissue differentiation in the axial skeleton
Source: Sci Rep. 2020 Dec 7;10:21364. doi: 10.1038/s41598-020-78206-4 (PMC7721728; doi:10.1038/s41598-020-78206-4)
Supplement: Supplementary file 1 — Supplementary information. [file 41598_2020_78206_MOESM1_ESM.pdf]

Supplementary information for:

Canonical and noncanonical TGF-β signaling regulate fibrous tissue differentiation in the axial skeleton

Sade W. Clayton, Ga I Ban, Cunren Liu, and Rosa Serra#

# Correspondence to:
Rosa Serra, Ph.D.
Department of Cell Developmental and Integrative Biology
University of Alabama at Birmingham
660 MCLM, 1918 University Blvd.
Birmingham, Al 35294-0005
205-934-0842
rserra@uab.edu

Table S1. qPCR details for Figure1A. Data was normalized to HPRT and expression levels calculated relative to vehicle control.

Comparison	Fold Change	Confidence Intervals 65%	P-value	Result
SCX				
Control (Vehicle)	1	1		
Control vs T_2hr	15.086	12.742 - 18.183	0.0001	UP
Control vs T_8hr	18.586	15.587 - 22.730	0.0001	UP
FMOD				
Control (Vehicle)	1	1		
Control vs T_2hr	1.462	0.282 - 11.520	0.609	-
Control vs T_8hr	20.604	15.005 - 28.415	0.0001	UP
ADAMTSL2				
Control (Vehicle)	1	1		
Control vs T_2hr	1.282	0.220 - 10.591	0.782	-
Control vs T_8hr	8.560	3.884 - 18.177	0.0001	UP

Table S2. qPCR details for Figure1B. Data was normalized to HPRT and expression levels calculated relative to vehicle control.

Comparison	Fold Change	Confidence Intervals	P-value	Result
		65%		
SCX				
Control (Vehicle)	1	1		
Control vs T	15.086	12.742 - 18.183	0.0001	UP
Control vs CHX	1.820	1.430 - 2.236	0.0001	UP
Control vs T/CHX	8.554	5.799 - 10.936	0.0001	UP
CHX vs T/CHX	4.700	3.192 - 6.453	0.0001	UP
T vs T/CHX	0.567	0.396 – 0.734	0.0001	DOWN

Table S3. qPCR details for Figure1C. Data was normalized to HPRT and expression levels calculated relative to vehicle control.

Comparison	Fold Change	Confidence Intervals	P-value	Result
		65%		
FMOD				
Control (Vehicle)	1	1		
Control vs T	20.604	15.005 - 28.415	0.0001	UP
Control vs CHX	0.356	0.249 - 0.487	0.0001	DOWN
Control vs T/CHX	0.495	0.374 - 0.636	0.0001	DOWN
CHX vs T/CHX	1.388	1.028 - 1.928	0.016	UP
ADAMTSL2				
Control (Vehicle)	1	1		
Control vs T	8.560	3.884 - 18.177	0.0001	UP
Control vs CHX	1.102	0.481 - 1.972	0.694	-
Control vs T/CHX	2.402	1.101 - 3.907	0.005	UP
CHX vs T/CHX	2.180	1.521 - 3.301	0.0001	UP

Table S4 qPCR details for Figure 2F. SCX, FMOD, ADAMTSL2 or PRG4 data was normalized to HPRT, expression levels calculated relative to TGFβ treated.

Comparison	Fold Change	Confidence Intervals	P-value	Result
		65%		
SCX				
TGFβ	1	1		
T vs T/SIS3 5ug/ml	1.314	0.056 - 29.668	0.624	-
T vs T/SIS3 10ug/ml	1.201	0.064 - 21.577	0.709	-
FMOD				
TGFβ	1	1		
T vs T/SIS3 5ug/ml	0.783	0.679 - 0.901	0.034	DOWN
T vs T/SIS3 10ug/ml	0.565	0.440 - 0.754	0.0001	DOWN
ADAMTSL2				
TGFβ	1	1		
T vs T/SIS3 5ug/ml	0.757	0.666 - 0.861	0.000	DOWN
T vs T/SIS3 10ug/ml	0.488	0.368 - 0.647	0.003	DOWN
PRG4				
TGFβ	1	1		
T vs T/SIS3 5ug/ml	0.610	0.409 - 1.017	0.144	-
T vs T/SIS3 10ug/ml	0.455	0.242 - 0.860	0.048	DOWN

Table S5. qPCR details for Figure 2J. SCX or ADAMTSL2 data was normalized to HPRT, expression levels calculated relative to vehicle control.

Comparison	Fold Change	Confidence Intervals	P-value	Result
		65%		
SCX				
Control (Vehicle)	1	1		
Ad-GFP vs Ad-DNSmad2	1.192	0.824 - 1.740	0.366	-
Ad-GFP vs Ad-GFP/T	4.105	3.147 - 5.463	0.002	UP
Ad- DNSmad2 vs Ad-DNSmad2/T	4.769	2.667 - 6.743	0.004	UP
Ad-GFP/T vs Ad-DNSmad2/T	1.162	0.769 - 2.287	0.743	-
ADAMTSL2				
Control (Vehicle)	1	1		
Ad-GFP vs Ad-DNSmad2	0.868	0.622 - 1.240	0.449	-
Ad-GFP vs Ad-GFP/T	9.758	5.950 - 17.070	0.000	UP
Ad- DNSmad2 vs Ad-DNSmad2/T	4.066	2.219 - 8.230	0.010	UP
Ad-GFP/T vs Ad-DNSmad2/T	0.417	0.182 - 0.846	0.040	DOWN

Table S6. qPCR details for Figure 2M. SCX, FMOD, ADAMTSL2 and PRG4 data was normalized to HPRT, expression levels calculated relative to vehicle control.

Comparison	Fold Change	Confidence Intervals 65%	P-value	Result
SCX				
Control (Vehicle)	1	1		
Ad-GFP vs Ad-Smad3	1.879	1.347 - 2.598	0.059	-
Ad-GFP vs Ad-GFP/T	8.999	6.387 - 13.927	0.000	UP
Ad-GFP vs Ad-Smad3/T	12.000	7.304 - 18.210	0.002	UP
Ad-GFP/T vs Ad-Smad3/T	1.334	0.769 - 2.162	0.259	-
FMOD				
Control (Vehicle)	1	1		
Ad-GFP vs Ad-Smad3	0.974	0.740 - 1.343	0.908	-
Ad-GFP vs Ad-GFP/T	3.531	2.428 - 5.268	0.000	UP
Ad-GFP vs Ad-Smad3/T	4.597	2.894 - 7.265	0.002	UP
Ad-GFP/T vs Ad-Smad3/T	1.302	0.827 - 1.890	0.202	-
ADAMTSL2				
Control (Vehicle)	1	1		
Ad-GFP vs Ad-Smad3	1.288	0.864 - 1.070	0.323	-
Ad-GFP vs Ad-GFP/T	23.292	16.119 - 35.690	0.018	UP
Ad-GFP vs Ad-Smad3/T	25.302	15.888 - 41.358	0.002	UP
Ad-GFP/T vs Ad-Smad3/T	1.086	0.561 - 2.105	0.602	-
PRG4				
Control (Vehicle)	1	1		
Ad-GFP vs Ad-Smad3	1.057	0.894 - 1.276	0.729	-
Ad-GFP vs Ad-GFP/T	3.620	3.079 - 4.336	0.002	UP
Ad-GFP vs Ad-Smad3/T	15.197	10.946 - 21.443	0.011	UP
Ad-GFP/T vs Ad-Smad3/T	4.199	3.146 - 5.498	0.022	UP

Table S7. qPCR details for Figure 4C. SCX, FMOD and ADAMTSL2 data was normalized to HPRT, expression levels calculated relative to TGFβ treated.

Comparison	Fold Change	Confidence Intervals 65%	P-value	Result
SCX				
TGFβ	1	1		
T vs T/BIRB 1ug/ml	1.208	0.227 - 6.467	0.586	-
T vs T/ BIRB 5ug/ml	1.221	0.212 - 7.246	0.568	-
T vs T/ BIRB 10ug/ml	1.362	0.214 - 9.20	0.552	-
FMOD				
TGFβ	1	1		
T vs T/ BIRB 1ug/ml	1.305	0.193 - 7.117	0.585	-
T vs T/ BIRB 5ug/ml	1.182	0.180 - 7.674	0.669	-
T vs T/ BIRB 10ug/ml	1.082	0.159 - 8.394	0.854	-
ADAMTSL2				
TGFβ	1	1		
T vs T/ BIRB 1ug/ml	1.095	0.224 - 5.732	0.688	-
T vs T/ BIRB 5ug/ml	1.034	0.191 - 5.713	0.843	-
T vs T/ BIRB 10ug/ml	0.990	0.179 - 5.871	0.936	-

Table S8. qPCR details for Figure 4F. SCX, FMOD and ADAMTSL2 data was normalized to HPRT, expression levels calculated relative to TGFβ treated.

Comparison	Fold Change	Confidence Intervals 65%	P-value	Result
SCX				
TGFβ	1	1		
T vs T/MK 1ug/ml	1.451	1.043 - 2.089	0.196	-
T vs T/ MK 5ug/ml	1.507	0.868 - 2.500	0.214	-
FMOD				
TGFβ	1	1		
T vs T/ MK 1ug/ml	1.258	1.048 - 1.580	0.123	-
T vs T/ MK 5ug/ml	1.338	1.110 - 1.514	0.016	UP
ADAMTSL2				
TGFβ	1	1		
T vs T/ MK 1ug/ml	1.243	1.000 - 1.474	0.196	-
T vs T/ MK 5ug/ml	1.066	0.686 - 1.458	0.681	-

Table S9. qPCR details for Figure 5C. SCX, FMOD and ADAMTSL2 data was normalized to HPRT, expression levels calculated relative to TGFβ treated.

Comparison	Fold Change	Confidence Intervals 65%	P-value	Result
SCX				
TGFβ	1	1		
T vs T/PD 1ug/ml	0.821	0.192 - 3.300	0.684	-
T vs T/PD 5ug/ml	0.251	0.068 - 1.900	0.038	DOWN
T vs T/PD 10ug/ml	0.119	0.035 - 0.324	0.000	DOWN
FMOD				
TGFβ	1	1		
T vs T/PD 1ug/ml	0.871	0.191 - 3.985	0.769	-
T vs T/PD 5ug/ml	0.259	0.047 - 1.574	0.045	DOWN
T vs T/PD 10ug/ml	0.174	0.036 - 0.681	0.007	DOWN
ADAMTSL2				
TGFβ	1	1		
T vs T/PD 1ug/ml	0.968	0.063 - 18.838	0.934	-
T vs T/PD 5ug/ml	0.266	0.049 - 1.450	0.034	DOWN
T vs T/PD 10ug/ml	0.119	0.021 - 1.205	0.006	DOWN

Table S10. qPCR details for Figure 5D. SCX data was normalized to HPRT, expression levels calculated relative to vehicle control.

Comparison	Fold Change	Confidence Intervals	P-value	Result
		65%		
EBF1				
Control (Vehicle)	1	1		
Control (Vehicle) vs T	0.589	0.418 - 0.834	0.000	DOWN
T vs T/PD 1ug/ml	1.067	0.983 - 1.159	0.518	-
T vs T/PD 5ug/ml	0.757	0.698 - 0.824	0.161	-
T vs T/PD 10ug/ml	0.994	0.519 - 1.916	0.663	-
CMAF				
Control (Vehicle)	1	1		
Control (Vehicle) vs T	0.622	0.556 - 0.696	0.000	DOWN
T vs T/PD 1ug/ml	0.863	0.717 - 1.052	0.323	-
T vs T/PD 5ug/ml	0.866	0.773 - 0.972	0.494	-
T vs T/PD 10ug/ml	0.842	0.657 - 1.092	0.331	-

Table S11. qPCR details for Figure 6B. SCX data was normalized to HPRT, expression levels calculated relative to Scrambled SiRNA control.

Comparison	Fold Change	Confidence Intervals	P-value	Result
		65%		
SCX				
Scrambled siRNA	1	1		
Scrambled siRNA vs Scx siRNA	0.189	0.121 - 0.270	0.0001	DOWN

Table S12. qPCR details for Figure 6D. SCX, FMOD and ADAMTSL2 data was normalized to HPRT, expression levels calculated relative to Scrambled SiRNA control.

Comparison	Fold Change	Confidence Intervals	P-value	Result
		65%		
FMOD				
Scrambled siRNA	1	1		
Scrambled siRNA vs T/ Scrambled siRNA	3.754	1.163 - 10.236	0.002	UP
Scrambled siRNA vs Scx siRNA	0.524	0.345 - 0.978	0.020	DOWN
Scx siRNA vs T/ Scx siRNA	2.155	0.815 - 5.054	0.074	-
ADAMTSL2				
Scrambled siRNA	1	1		
Scrambled siRNA vs T/ Scrambled siRNA	3.462	1.012 - 11.618	0.029	UP
Scrambled siRNA vs Scx siRNA	0.998	0.369 - 3.397	0.999	-
Scx siRNA vs T/ Scx siRNA	2.148	0.480 - 7.983	0.203	-

Table S13. Primer sequences for qPCR in alphabetical order.

Gene Name	Abbreviation	Forward Primer: 5'-3'	Reverse Primer: 5'-3'
Adamtsl2	*Adamtsl2*	GGG-CAA-CAA-TCA-TCT-TGG-TTA-CT	CCG-TCG-GTA-CTT-GAC-CAC-T
C-Maf Proto-Oncogene	*cMaf*	GCT-TCA-GAA-CTG-GCA-ATG-AA	GTC-TCC-ACC-GGT-TCC-TTT-TT
EBF Transcription Factor 1	*Ebf1*	GCA-TCC-AAC-GGA-GTG-GAA-G	GAT-TTC-CGC-AGG-TTA-GAA-GCC
Fibromodulin	*Fmod*	GGG-GTC-ACC-CAA-GCT-GCT-GT	TGA-CGT-CCA-CCA-CCG-TGC-AG
Hypoxanthine Phosphoribosyltransferase	*Hprt*	TCA-GTC-AAC-GGG-GGA-CAT-AAA	GGG-GCT-GTA-CTG-CTT-AAC-CAG
Proteoglycan 4	*Prg4*	GAA-AAT-ACT-TCC-CGT-CTG-CTT-GT	ACT-CCA-TGT-AGT-GCT-GAC-AGT-TA
Scleraxis	*Scx*	ACT-CTT-CAG-TGG-CAT-CCA-CCT-TCA	TCT-GCC-TCA-GCA-ACC-AGA-GAA-AGT

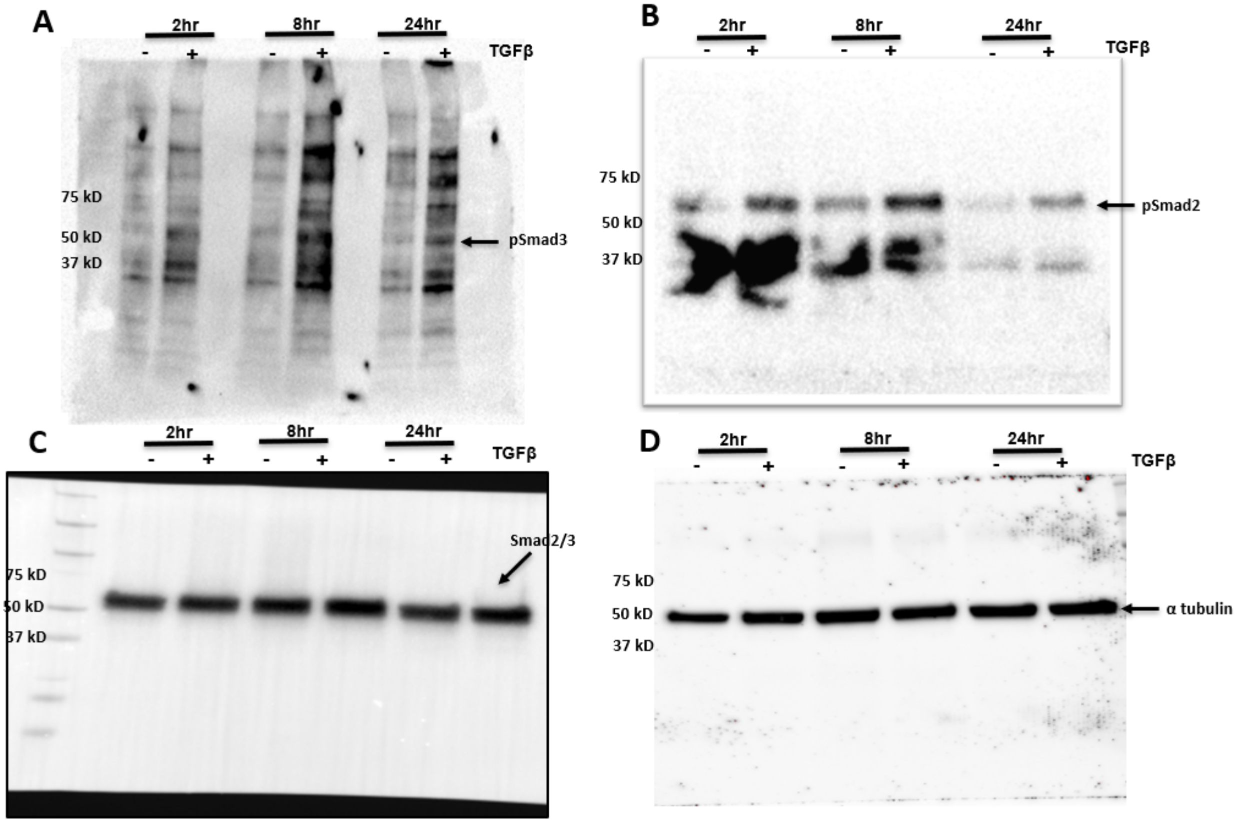

Figure S1. Example of uncropped (A) pSmad3 (B) pSmad2 (C) Smad2/3 and (D) α tubulin western blot from Figure 2, panel A

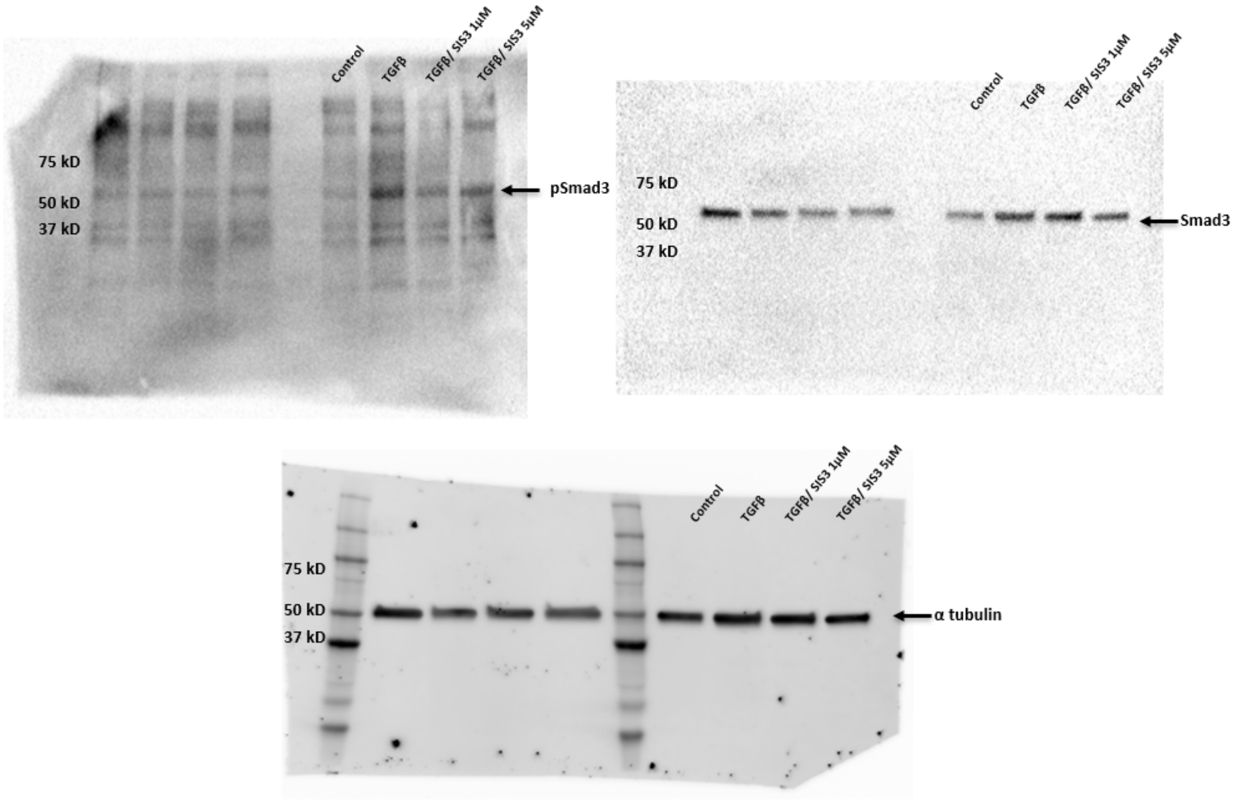

Figure S2. Example of uncropped (A) pSmad3 (B) Smad3 and (C) α tubulin western blot from Figure 2, panel D.

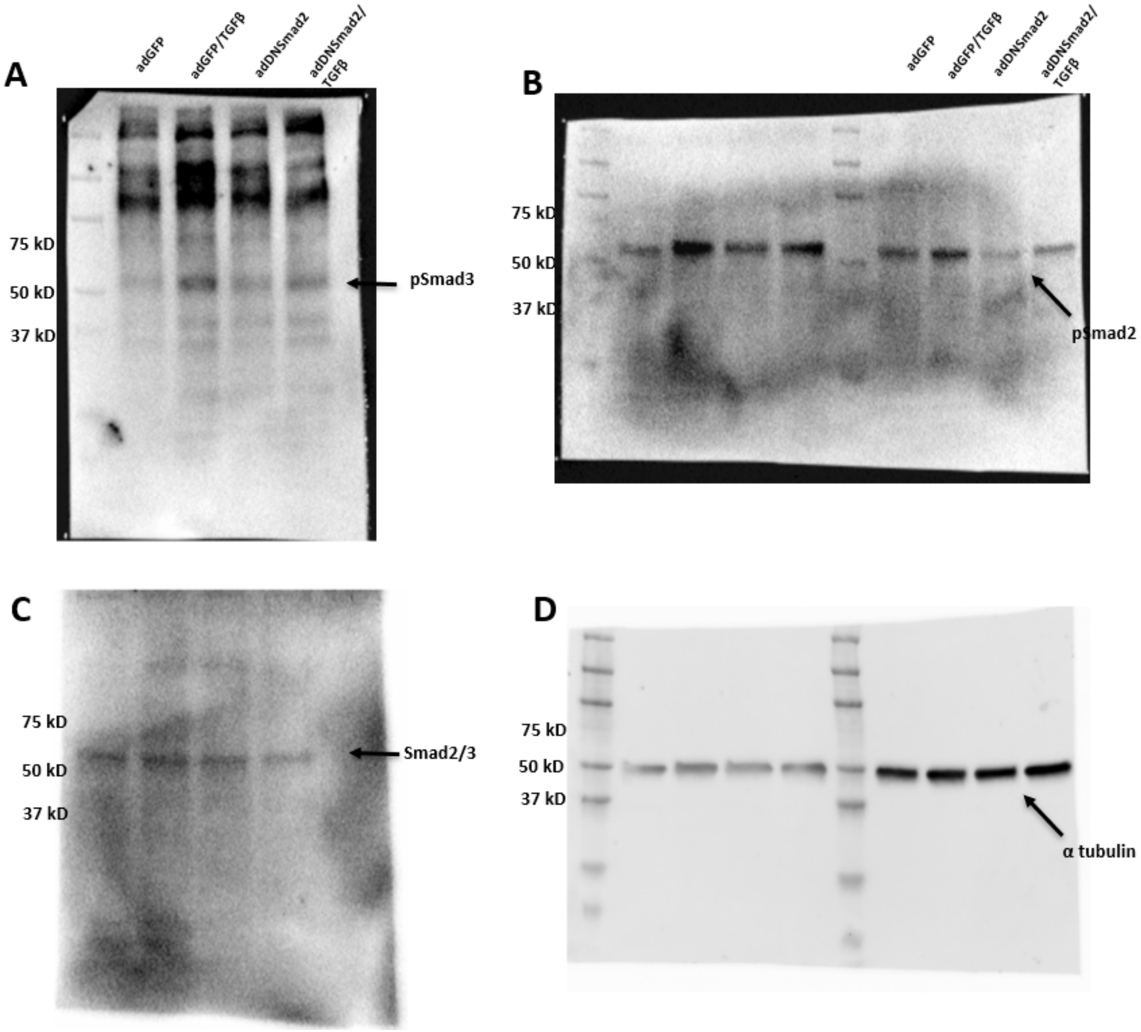

Figure S3. Example of uncropped (A) pSmad3 and (B) pSmad2 (C) Smad2/3 and (D) α tubulin western blot from Figure 2, panel G

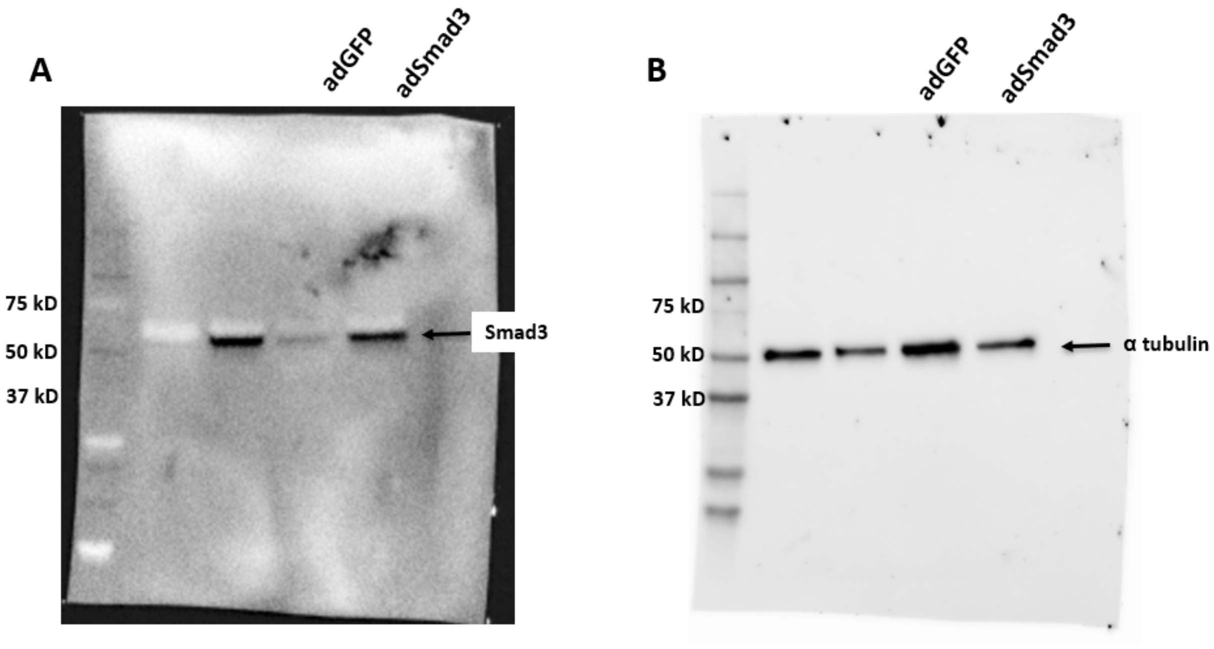

Figure S4. Example of uncropped (A) Smad3 and (B) α tubulin western blot from Figure 2, panel K.

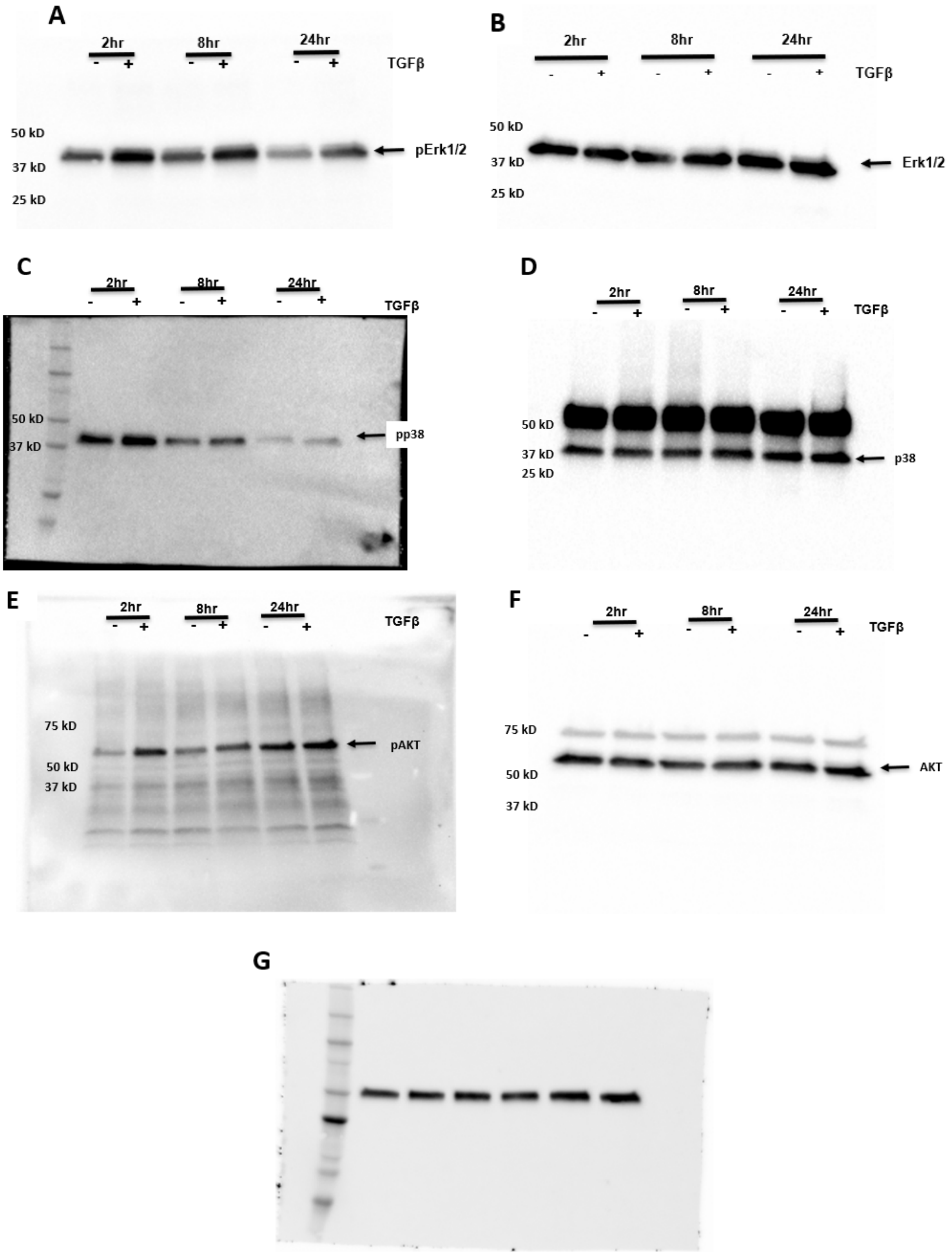

Figure S5. Example of uncropped (A) pErk1/2 (B) Erk (C) pP38 (D) P38 (E) pAKT (F) AKT and (G) α tubulin western blot from Figure 3, panel A.

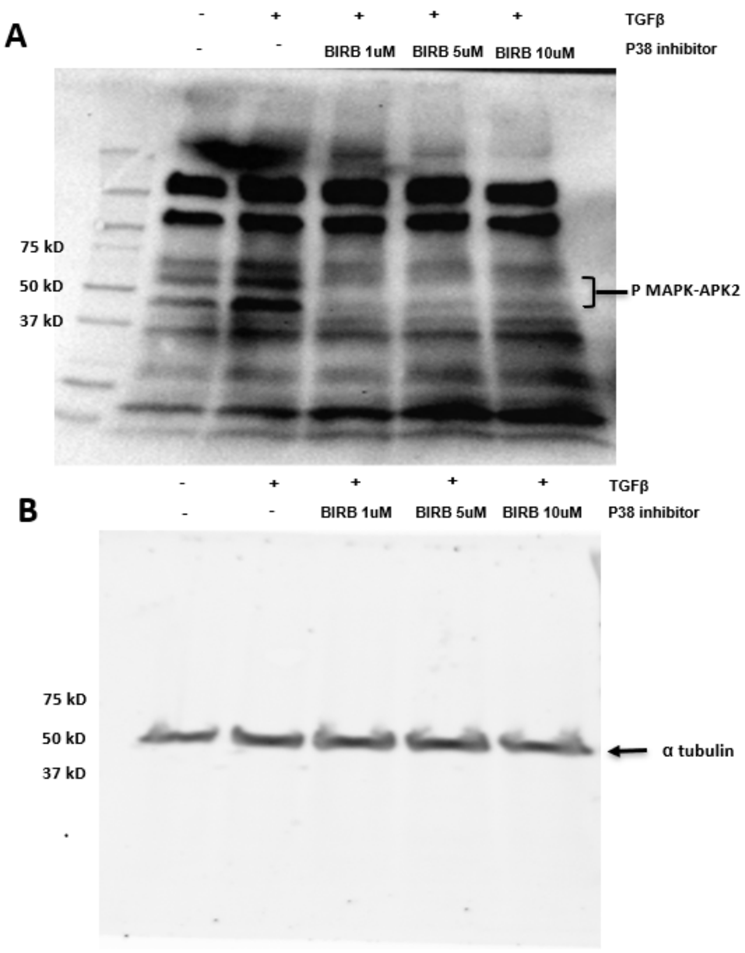

Figure S6. Example of uncropped (A) pMAPK-APK2 and (B) α tubulin western blot from Figure 4, panel A

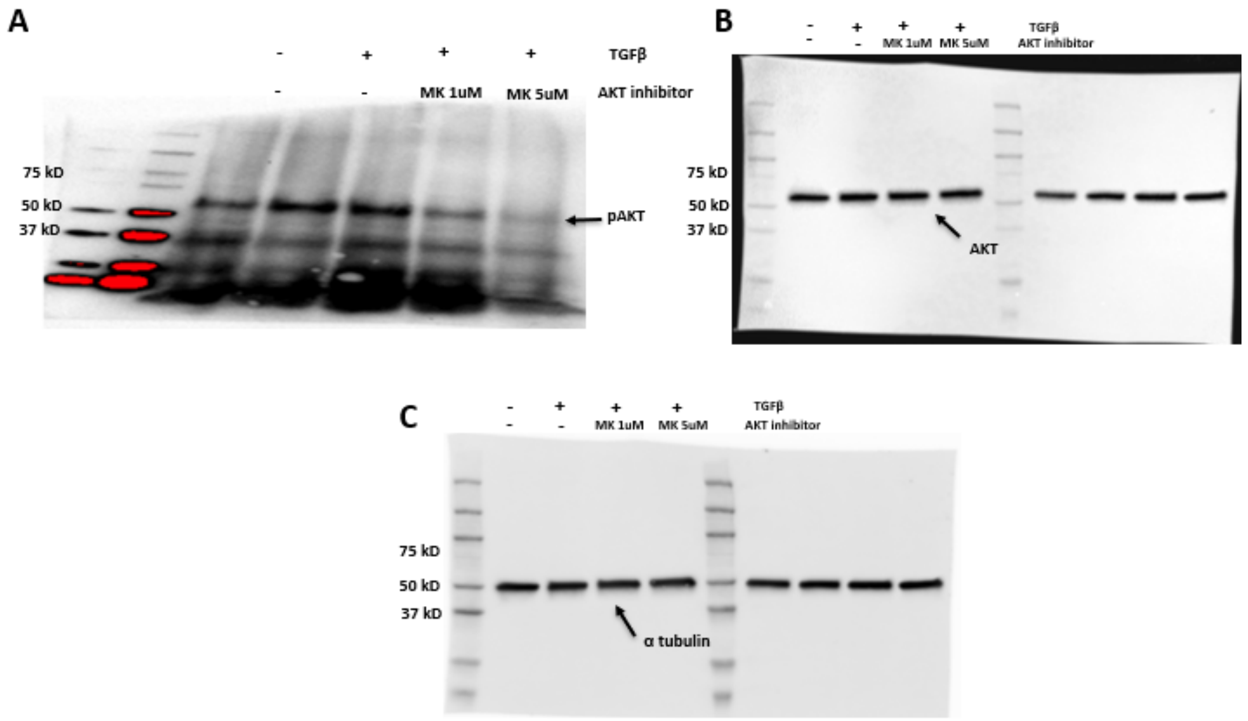

Figure S7: Example of uncropped (A) pAKT (B) AKT and (C) α tubulin western blot from Figure 4, panel D.

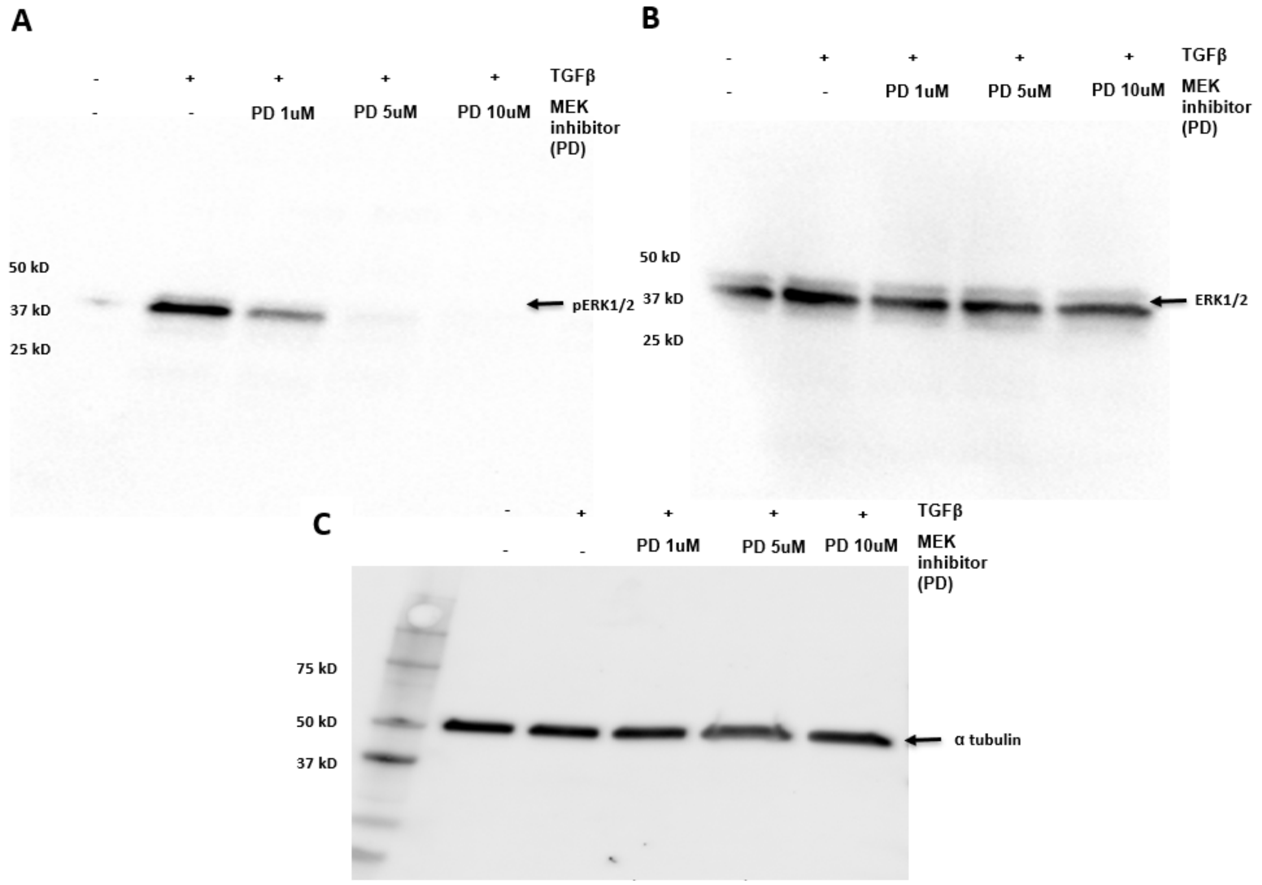

Figure S8: Example of uncropped (A) pERK1/2 (B) ERK1/2 and (C) α tubulin western blot from Figure 5, panel A.

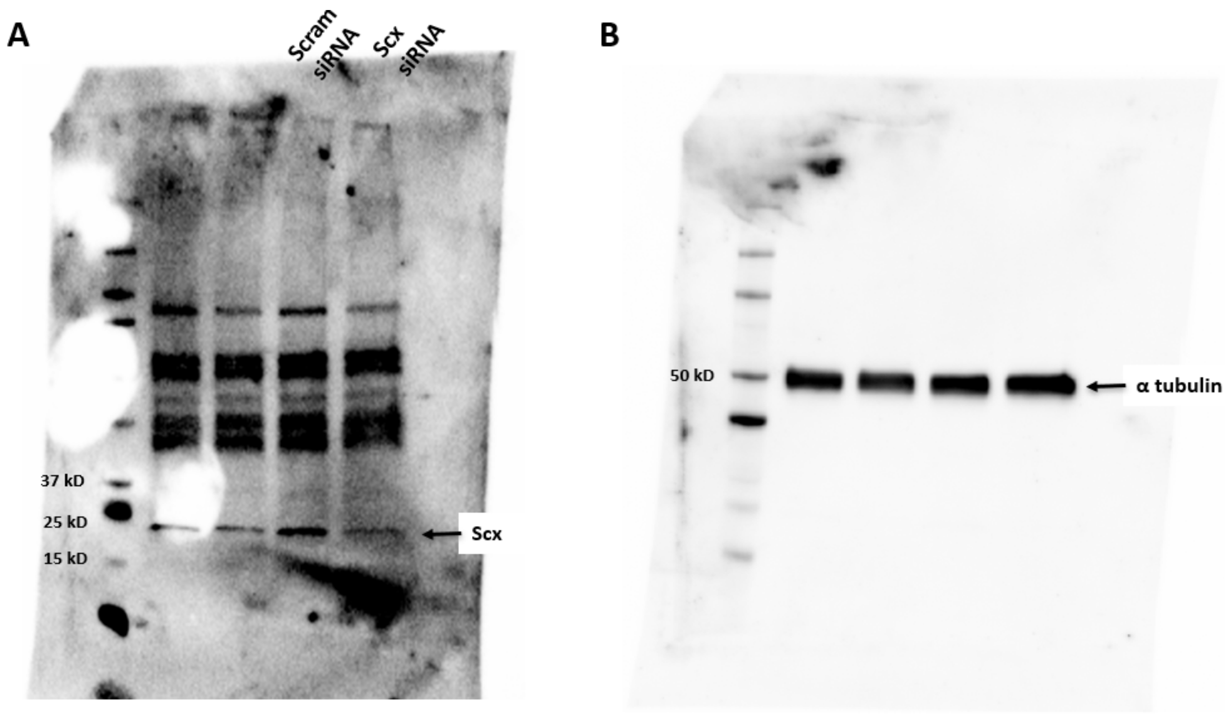

Figure S9: Example of uncropped (A) Scx and (B) α tubulin western blot from Figure 6, panel A.
